# ‘On the Perimeter of the Lawful’: Enduring Illegality in the Irish Family Planning Movement, 1972-1985

**DOI:** 10.1111/jols.12055

**Published:** 2017-12

**Authors:** Emilie Cloatre, Máiréad Enright

**Affiliations:** * Kent Law School, University of Kent, Canterbury, Kent CT2 7NZ, England; ** Birmingham Law School, University of Birmingham, Edgbaston, Birmingham B15 2TT, England

## Abstract

Between 1935 and 1985, Irish law criminalized the sale and importation of condoms. Activists established illegal markets to challenge the law and alleviate its social consequences. They distributed condoms through postal services, shops, stalls, clinics, and machines. Though they largely operated in the open, their activities attracted little direct punishment from the state, and they were able to build a stable network of medical and commercial family planning services. We use 30 interviews conducted with former activists to explore this history. In doing so, we also examine the limits of ‘illegality’ in describing acts of everyday resistance to law, arguing that the boundaries between legal and illegal, in the discourses and practices of those who sought to challenge the state, were shifting and uncertain. In turn, we revisit ‘illegality’, characterizing it as an assemblage of varying selectively-performed political practices, shaped by complex choreographies of negotiation between state and non-state actors.

Between 1935 and 1985, Irish law^1^ criminalized the sale and importation of condoms and other contraceptives. This prohibition was introduced by the Criminal Law Amendment Act^2^ and reflected an effort on the part of the fledgling Irish state to enforce Catholic social mores.^3^ In the 1970s, activists began to attack the 1935 ban on sale and importation of contraception. From 1971, liberal lawmakers had been proposing law reform bills in Parliament with little success.^4^ In 1973, in *McGee* v. *AG*,^5^ a married woman challenged the seizure of contraceptives which she had imported from England by post, in violation of the 1935 Act. The Supreme Court accepted that the prohibition on importation violated the constitutional right to marital privacy; in the process, it recognized a limited constitutional right to access contraception, for married couples.^6^ However, conservative parliamentarians ensured that legislation to give effect to this right was slow in coming. This story of difficult institutional law reform has been told before.^7^ However, in focusing on that story, others are often marginalized. At a time when formal mechanisms of law reform had either stalled, or were only accessible to conservative official actors, a network of activists organized to circumvent the law. Family Planning Services Ltd. (FPS), local family planning clinics, the Irish Family Planning Association (IFPA), and Well Woman established illegal markets to challenge the contraceptive ban and alleviate its social consequences. They imported condoms in bulk, initially distributing them through postal services and later through shops, stalls, clinics, and machines. This article tells the story of those networks, and their endurance in conditions of illegality. As well as contributing to existing scholarship on the history of contraceptive movements both within and beyond Ireland, it draws on activists’ stories to provide a rich sense of how those working to resist a law they oppose may experience the ‘illegality’ of their cherished projects.

It is important to clarify the general sense in which we use the word ‘illegal’ in this article. The law was widely understood to criminalize the sale of condoms, in some way, at all times in the period under study. Until 1980, sale was absolutely prohibited. In turn, importation for sale was illegal. Before 1980, rather than selling condoms outright, activists supplied them in exchange for a ‘donation’; they thus exploited a ‘loophole’ in the letter of the law. Their activities were strictly unlawful, in the sense of circumventing the law’s purpose, rather than illegal in the sense of being explicitly prohibited by it. In 1980, the Health (Family Planning) Act 1979 came into force. Sale remained a crime where condoms were sold to the wrong person, or by the wrong person or in the wrong location. The new Act restricted legal sale to those who had obtained a doctor’s prescription, and bought from pharmacists – usually married couples who were using contraceptives for family planning purposes.^8^ This restriction applied to non-medical contraceptive devices such as condoms, as well as to pharmaceuticals such as the contraceptive pill. Supply otherwise than by sale, remained an offence.^9^ The ‘donation’ loophole was comprehensively closed, and family planning distributors’ activities became bluntly illegal. It was not until 1985 that the Act was amended to allow family planning clinics to sell and supply condoms without prescription, to both married and unmarried adults.^10^ Thus, in the early 1980s, the family planning network’s attention turned to challenging and circumventing this new arrangement, fighting for the more open distribution model they had spent years establishing.

Shifts in condoms’ black-letter legal status certainly had consequences for activists’ strategies and sense of their own position in relation to the law. However, this internal technical shift is only a small part of the story of illegality here. A constant feature of activists’ experience was a sense of being in a position of transgressive marginality in relation to the law, which affected both their self-perception and their positionality in Irish society. It is that complex ‘outlaw’ position that we explore, under the shorthand of ‘illegality’. Often even our informants described their experience in terms of simple vernacular dichotomies between what was ‘legal’ and what was not. At the same time, the stories they told upset those very binaries. These are stories of inhabiting illegality. They demonstrate that illegality is experienced as an assemblage of uncertain, shifting, varying, and selectively-performed political practices, constantly shaped by complex choreographies of negotiation between state and non-state actors.

## Study and Methods

The article is based on 30 interviews with people active in the distribution of condoms in Ireland from 1972 to 1985. Most were members of FPS, the IFPA, the Irish Family Planning Rights Association (IFPRA), or the early family planning clinics. Others were involved in the radical feminist Contraceptive Action Programme (CAP), the Well Woman Clinic, and the student movement. Some informants were nationally recognized campaigners in these organizations, while others’ work was out of the public eye, as service providers, lawyers, and medical personnel. We identified an initial sample of participants from research in newspaper, legal, and organizational archives. Once we had contacted (by email or phone) and met those initial participants, they recommended other key actors in their former networks that we followed through. This method had some limitations; in particular, we were partly relying on surviving friendship networks among key actors which meant that we were less likely to interview less prominent members of the movement. Our loosely-structured interviews were inspired by ethnographic methods of inquiry. Although we had broad topics in mind, we often let the conversation flow as personal memories unfolded. While paying particular attention to questions of law, and often pressing interviewees to think more about issues of law, we retained a focus on participants’ everyday experience. We supplemented the interviews with research in newspaper archives and relevant academic literature, legislation, and court cases. We also accessed the archives of Dr. Derek Freedman of FPS at University College Dublin (UCD), and those of Attic Press at University College Cork (UCC), which contain material relating to the Irish women’s movement. Some participants gave us access to small personal collections of activist ephemera. As with most projects of this kind, difficulties were presented by the passage of time. Most organizations studied did not preserve a complete archive. We were reliant in many respects on informants’ memories of their time as activists. This presented some shortcomings: for example, most informants were unwilling to rehearse past conflicts, or their memories were coloured by subsequent involvement in other projects. The passage of time also presented opportunities: informants could speak openly about past engagement in illegal activities without fear of reputational damage or other consequences. In the decades since these illegal activities took place, the family planning movement has come to be celebrated, so that informants were proud to be telling their stories.

A number of our informants were members of both FPS and the IFPA. In this article we pay more specific attention to FPS for several reasons. First, we were able to obtain better first-person access to almost all the founders of FPS, as well as to relevant archives, allowing us to construct a richer, more coherent narrative. Second, FPS initiated and became the centre of a mass condom-distribution network,^11^ of which the IFPA, like the clinics and other organizations mentioned, subsequently became a customer member. In telling the story of condom distribution specifically, rather than of family planning policy formation more broadly, FPS emerges as a central actor. Finally, the IFPA receives far more attention in the available literature on the Irish family planning movement;^12^ we were interested in exploring a less-told story. Of course, one reason why the IFPA may receive more attention is that its modes of activism are perceived to fit more neatly with a liberal law reform narrative: it was led by doctors from the beginning, and was understood – at least in the period under study – to be more reluctant to initiate law-breaking activity.^13^ While such contrasts between strategies for law breaking and strategies to maintain respectability are a running theme in birth-control literature, we do not mean to erect a binary between the ‘radical’ disobedience of FPS and the ‘responsible’ disobedience of the IFPA.^14^ However, because of their greater willingness to break the law during the period under study, FPS’s story was a more fruitful one for our inquiry.

The history of FPS feeds into broader scholarship that has explored the history of birth-control movements. There, others have, in different national contexts, explored the entangled personal, social, and political trajectories that shaped the transformation of birth control from socially unacceptable practices to everyday devices, and the adjacent transformations of states’ approaches to sexuality and sexual health.^15^ Surprisingly, the history of the birth-control movement in Ireland has been given relatively limited attention so far. By looking closely at the experience of one of its key organizations, we seek to address this gap in knowledge, while also reflecting on the broader contributions of this history to discussions about the nature of illegality as lived experience.^16^

## Early Days

FPS began as a condom mail-order service in Dublin in 1972. Their model built on stable cracks within the legal order.^17^ It was common knowledge that individuals often imported small amounts of contraceptives when returning from England, Northern Ireland, and elsewhere.^18^ The Irish Women’s Liberation Movement made a spectacle of this practice in 1971, with the contraceptive train, encountering no resistance from customs officers.^19^ The Fertility Guidance Company (later the IFPA) had been operating for almost four years but did not distribute contraceptives beyond their own clinic. Instead, they gave prescriptions and order forms to clients who imported items on their own, ordering by post from the International Planned Parenthood Federation in England. Poor clients were given free contraceptives, imported in small batches by clinic staff and supporters.^20^ FPS’s founders determined that contraceptive distribution should also take place outside the clinics: ‘kind of, four or five of us, half a dozen of us decided the next logical step was to start distributing non-medical contraceptives.’^21^

At first, FPS was a very small scale, almost ‘personal’, operation. The group fulfilled orders themselves from their homes, improvising a weekly rhythm of labour:

We had no money, no money at all apart from we put I think a fiver a head to buy stamps and envelopes and sellotape … and we met every Thursday in one of our houses … The orders would come in, we would dispatch them every Thursday around somebody’s kitchen table and lick envelopes and put on stamps and address envelopes and send them off, bank the money, the next Thursday the same thing again and then it grew from that …^22^

With a private £500 loan, and a £500 Rowntree Foundation grant, they purchased contraceptives from the London Rubber Company (now Durex).^23^ London Rubber delivered FPS’s goods to a friendly pharmacist in Portadown in Northern Ireland. He was paid to store the deliveries in his garage. Several people did delivery runs as ‘couriers’ from the garage to Dublin; including a commercial traveller for a pharmaceutical company.^24^ One member of FPS travelled over the border in a small van, as often as he could, to collect supplies, smuggling the orders back in boxes. Members stored boxes in their homes:^25^

So [he] comes down with his carload of condoms … I think the person who was doing it next week took them home, you see. So you are ‘on’ this week, so they are all in your house. OK so [he] now brings them in and there they are boxes of Durex and whatnot, and sure enough the first week we had – it doesn’t matter – 20 orders. It doesn’t matter what it was. Actually it was something; ‘Good God, 20’. So you open them and they are sitting round the dining room table, and I open them and there is one from somebody in Athlone and they send a pound and they want a dozen Durex. I don’t know how they reached them but OK the pricelist started going, the list started to go out to people or people would write in and they’d just say ‘What have you?’ and we’d send them a list of everything.^26^

If supplies ran low a member might bring back contraceptives when travelling abroad.^27^ Customers’ orders came to a Dublin post office box. One member, who had worked with the postal service, arranged it. Interested journalists published the box number in their newspaper articles. Customers would write with specific requests enclosing cash, and often telling personal stories. On Thursday evenings, from about 7pm until 11pm, FPS’s founders set up a kitchen table production line to fulfill the orders by post: one person would open the envelopes, another would read out the orders, a third would address the envelope, a fourth would pack it, a fifth stamp it and a sixth keep records.^28^ Their activities were often combined with domestic life. Some members were married couples. At least one member described involving their young children in fulfilling orders by packing contraceptives in envelopes.^29^

Condom sales were profitable. FPS soon amassed a significant amount of money,^30^ enabling them to expand to a small office in two rooms above a chemist shop in Leeson Street.^31^ They employed staff, just to handle incoming orders.^32^ Later they established a clinic in Pembroke Road, employing nurses, doctors, and an education officer. It eventually became the centre of a network of other distributors and clinics.^33^ FPS assisted groups who wanted to establish clinics outside Dublin, and were a source of advice as these clinics grew.^34^ Rural clinics were not officially affiliated to or governed by FPS. In most respects they operated independently. However, FPS initiated methods and repertoires of activity which others went on to replicate. The relationship between FPS and other clinics was generally characterized more by solidarity than by hierarchy. Relationships were also commercial.^35^ FPS became wholesalers of non-medical contraceptives to fledgling clinics, and sympathetic doctors and chemists all over the country; trained nurses and doctors in family planning; and supplied speakers for public events nationwide.^36^ FPS’s early development was characterized by rapid, improvised assembly. ‘We didn’t think too much about this when we started … but by the time the thing got up and running at such incredible speed, now we were responsible.’^37^ Founders spoke of this expansion, as reactive to customers’ needs, if not entirely unplanned. They found themselves ‘on a roller-coaster’.^38^

Galway Family Planning Clinic’s founders remembered similar makeshift beginnings. Initially, there were two groups in Galway; a mail-order service and a collective interested in establishing a clinic but unable to secure premises. The mail-order service began running, advertising in a local free newspaper, *The Galway Advertiser*, while the clinic project stalled.^39^ They used the kitchen table mail-order process devised by FPS, adapting their order form and price list.^40^ Customers were asked to include a stamped addressed envelope with their order, so that the service organizers did not have to pay for stamps.^41^ The process worked well, but soon it became clear that the clinic project would have to be revived:

We started to be worried that there was a lot of ignorance in the letters we were getting. We started to worry about the ethical value, the ethical issues involved in giving people something without giving them education. And this was increasingly, you know, not all, 90% of the letters were people who wanted condoms, but just the occasional letter came in, you said, ‘Oh, Christ, there’s so much ignorance that really we need to talk to these people before we give them’, so we thought how to do that. So we decided to meet with this group in Galway, you know, that had eminent people in it, doctors, proper people, so we went to see them. And essentially the equation was we had the trade and they had their own doctors and neither of us had a premises.^42^

Following a makeshift process not dissimilar to that followed in Dublin, Galway providers moved from kitchen tables to new offices: premises were obtained and staffed by unpaid volunteers,^43^ recruited by word of mouth through friendship networks.

## Precarity, Tactics, and the Conditions of Illegality

The condom distribution movement did not enjoy the same security as formally recognized medical agents. As a result, early organization was marked by what Massey calls ‘thrown-togetherness’: the processes of judgement, learning, and improvisation that take place in these sorts of precarious circumstances.^44^ The new family planning network was assembled piecemeal, and continued to operate through complex tactics and adjustments; its contours reflecting the resources available to those involved, and the myriad consequences of the law’s approach to contraception. Acting against the law had a number of direct consequences on the service.

First, the illegality of condom sales, and accompanying moral discourses, affected potential customers. FPS’s founders remembered receiving letters which indicated customers’ humiliation:

Yeah and you know, that mail order service was extremely sad because on a Sunday after Mass, my wife would still go to Mass and she would come home and we would sit around the table afterwards. And we would have a bag of mail – now the tragedy of this was, the people who were sending the letters, felt that I was sitting in judgement of them, saying ‘Oh, well she can get three’, ‘You are entitled to five’ or you are not … in case we didn’t make a strong enough case. Which of course I wasn’t – but one of the greatest crimes I committed was not preserving those letters because they were heart-breaking and they were horrendous.^45^

Services had to adapt – for instance, distribution by mail ensured anonymity for those living far from a clinic or ashamed to buy condoms.^46^ Similarly, early visitors to the clinics could be ‘edgy’, ‘ashamed’, ‘uncertain of themselves’, ‘embarrassed’,^47^ ‘desperate’,^48^ or ‘abandoned’.^49^ Accordingly, confidentiality and sympathy were paramount.^50^

Second, the clinics’ association with illegality in the public consciousness generated difficulties in establishing services. Many began in unsuitable premises,^51^ because they could get nothing better.^52^ In Galway, the first clinic’s landlord ‘nearly had a nervous breakdown when he realised who he had rented it to’.^53^ Interviewees described those premises, above a garage on Raleigh Row, in the shadow of St Ignatius’ church, as a ‘hovel’ with rickety stairs,^54^ ‘the worst, shoddiest looking place in the world’.^55^ They agreed that, if possible, female volunteers should not work there alone.^56^ The taint of illegality, and the taint of immorality associated with contraceptives at the time, meant that finding sympathetic doctors was challenging in most places. For example, in Limerick, the first clinic struggled for a long time to find a doctor, making do with twice-weekly sessions organized by the IFPA.^57^ The Well Woman Clinic opened without a doctor.^58^ Nurses were easier to hire – they were typically mothers seeking part-time work.^59^

The precarity of the contraceptive movement’s activities was also a direct result of a pervasive sense that acting outside the law entailed the possibility of devastating enforcement – even if, as we return to below, this threat rarely materialized. The impression of a persistent ‘ambient insecurity’ surrounding family-planning activities,^60^ or as one clinic nurse put it, the sense that ‘illegality was in the air’,^61^ meant that activists developed careful tactics to negotiate their relationship with the law. Of course, and as we return to below, this sense of insecurity stemmed not only from the law, but also from other social and religious pressures: clinics were not just breaking the law, they were also breaking (some) moral codes of the time. To be able to persevere in their activities, they had to learn to diagnose, adapt to, and sometimes exploit the imposed precarity of their situation.^62^ The movement’s capacity to strategically negotiate the law’s apparent limits was crucial. They often used law’s own techniques against it in sophisticated ways, playing law as a ‘game’.^63^

FPS relied heavily on vernacular techniques of legal interpretation.^64^ Although they had some access to legal advice through friends, and later were in regular contact with their solicitors, FPS’s founders present themselves as interpreting the 1935 Act which prohibited the sale of condoms on their own:

Now, perhaps it was my … at the time I was training to be, I was becoming a statistician and that implied I had an analytical frame of mind so I started to look closely at the legislation and I decided that there was a loophole that nobody had ever spotted before.^65^

Notably, they read the Act as prohibiting selling condoms, but not supplying them in exchange for donations. They decided that:

basically, the law prevented the importation for sale and the selling … Nothing about usage and nothing about bringing them in in any other way apart from importing for sale. So you could in theory import as long as they weren’t for sale.^66^

Thus, FPS held themselves out, not as selling condoms, but as giving them away, while inviting voluntary donations to support their educational and other activities.^67^ If, in 1973, I wrote to FPS as a first-time customer to order condoms I would receive, along with my purchase, a folded ‘effective price list’,^68^ detailing the products available and suggesting an appropriate donation for each item. An FPS ‘delivery advice’ slip intended for wholesale customers in the year before the Act which partially legalized sale of contraceptives came into force bears the legend:

This document is not an invoice, nor a demand for payment, since the sale of contraceptives is illegal. The values shown above are for [FPS’s] use only, although they may also be of interest to clients. Any donations towards the expenses incurred by [FPS] will be greatly appreciated.^69^

The Galway mail-order service took a similar approach,^70^ feeling ‘safe’ in adopting a tested method.^71^ In time, the IFPA, formerly so wary of this strategy, followed suit.

This creative vernacular approach to legal interpretation later grounded FPS’s other activities. For instance, FPS began by importing contraceptives from Northern Ireland. The December 1973 Supreme Court decision in *McGee* v. *AG* 72 recognized married couples’ constitutional right to import contraceptives for their own personal use. Successive governments failed to legislate for this right for six years, during which the status of the 1935 Act’s prohibition importation was uncertain. FPS took advantage of this ambivalence. They decided that it was permissible, not only to import small amounts of condoms ‘for personal use’ in luggage or a car boot, but to import truckloads in bulk by ship, provided that they were not imported ‘for sale’. They did not wait for the state to confirm the validity of this interpretation:

Well we would say we are distributing them to individuals, we’re not selling them, so we’re following on the principle of McGee, just doing it in bulk. There’s nothing to say you can’t be a distributor for importation … So we fill out the customs forms and say these are for free distribution and they couldn’t do anything because if it was free distribution, it’s not illegal … We found a formula of words and we used that every time, the exact same formula of words.^73^

The donation ruse purified the sale of condoms, replacing a dangerous relationship with an altruistic one.^74^ In their interpretation of the statutory prohibition on sale, FPS relied on what Ewick and Silbey refer to as ‘rule literalism’: the subversion of a rule by rigid adherence to it. Such interpretation is not disobedient as such because the rule does not contemplate the particular action. Thus the challenge to the law remains ‘indecipherable and invulnerable to control’.^75^ Interviewees were aware that the sale/donation distinction was thin and formalistic – they were ‘going along with’ the law by using an ‘act’^76^ that enabled them to achieve their aims.^77^ Our interviewees slipped self-consciously between the language of gift/donation and sale/purchase. For instance, describing FPS’ first premises, one said:

You’ve got a counter so you walk in like you would to any shop and buy your stuff over the counter or some would give a contribution I mean OK you’d buy it over the counter.^78^

It was typical of FPS at the time that they did not rely on lawyers to develop this interpretative tactic:

We worked through the law and we read the law and most people had not read the law. I don’t think the lawyers had read the law, and what I mean by that is when we went in to meet our lawyers we were telling them what the law was.^79^

FPS founders acknowledged that their readings of the governing law were not mainstream, but they were willing to deploy them as necessary, and to assert their validity:

Well I think we were correct legally in what we were saying but we were following a particular interpretation of the law. If that had been challenged, it might not have been upheld.^80^

As non-lawyers, they sometimes found their approach clashed with that of their legal advisors, whose training directed them to read the law in more constrained ways. For instance, correspondence with a barrister in 1980, on possible readings of the 1979 Act partially liberalizing sale of contraceptives shows his frustration at their determination to circumvent the new Act using what he sees as ‘layman’s’ strategies. He responds with a long explanation of the principles of statutory interpretation, warning them that while their arguments ‘may be adequate debating points, they do not carry the same force when interpreting statute’.^81^

Perhaps paradoxically, the association of condoms with illegality also lent some resilience to the early family planning groups. It was central to their ‘market persistence’,^82^ simply because it made condoms so scarce. Indeed, once condoms were sold widely in pharmacies, many clinics suffered from the loss of a reliable income that allowed them to serve poorer clients for free^83^ and to support other organizations.^84^ At times, FPS were also able to boost this income by deliberately manipulating the sense that the supply of condoms was vulnerable to law enforcement. As one informant explained:

[T]here was regular legal scares of one sort or another, some of them we generated because we found that the effect that it would have that once there was some issue that we were threatened with being closed down … we’d get queues of people coming in the door looking to stock up and we’d get, our mail volume would go up substantially and didn’t go down again after the scare. So after a while we created a few scares because it was good marketing.^85^

## Plural and Overlapping Orders

Decisions to resist state law were made within multiple overlapping normative orders. In Ireland, the contraceptive legislation was rooted in a coimbrication of church teaching and state laws, and family planning clinics were established at a time when this nexus was becoming heavily contested.^86^ Activists were committed to a developing ethic^87^ which ran counter to the conservative political settlement, and allowed them to represent themselves as engineers of an emerging social path, rather than as rogue agents of a criminal alterity:

I don’t think we regarded the ideas that we were putting forward as outside mainstream ideas. They were the establishment but the establishment had ceased to be mainstream.^88^

The law, on this view, no longer deserved obedience.

Many interviewees were from the Protestant minority in Ireland, and so were used to – if often frustrated by – circumventing the strict teachings of the majority church. Others came from Catholic families which took a resistant approach to church edicts, or had themselves already transgressed them; for instance, by ending a marriage. Some were foreigners or had lived in countries where contraception was readily accessible. Most were well-educated, beneficiaries of free secondary school education and widening access to universities. Some had been active in the radical student movement, the women’s movement, and broader civil rights struggles. Interviewees also drew a sense of counter-legitimacy for their activities from their experience of engaging with customers. They knew that they were providing a benefit to people who could not otherwise obtain it.^89^ They were conscious of the burdens which the law visited upon women. Many women activists were galvanized by their own difficulties in accessing contraception, or by their mothers’ difficult experiences in rearing large families.^90^ A founder member of FPS spoke in these terms:

I thought it was a women’s rights issue, that she didn’t have to become pregnant every time she had sex, that is all. Because I was pissed off at having five children myself. I had three children under two and a half.^91^

Many were conscious that they were addressing a prevailing inequality of power in Irish society, that led them to see the law as driven by hypocrisy:

You see, that’s the farce of the thing, that’s why we kept saying it was ridiculous because, like, if you knew what you were doing you could get them easily. Well, not easily, but you could get them. You know, most of the cabinet and the government had got them easily you know, and that was ridiculous. But meanwhile, in sort of working class areas, people didn’t even know where the family planning clinic was or that it existed or would go in there, you know, because it was shameful.’^92^

For some, this distinguished their projects from unsavory activities: ‘you were respectable in terms of, you weren’t trying to “do” people, you were simply trying to change the law.’^93^

However as we mentioned earlier, activists’ work was also inevitably framed by the context in which they were operating, itself largely determined by Irish social and religious norms of the time. Indeed, by virtue of their public alterity, they came under pressure from a range of religious sources seeking to defend the prevailing conservative consensus. The primary deterrents against condom distribution came not from law but from pervasive moral and religious norms, themselves diffuse, unevenly distributed, and subject to challenge. The church hierarchy commonly intervened, denouncing organizations and naming individuals in an attempt to shame them. Religious activists also engaged in more low-key, day-to-day interventions, from picketing to praying outside the clinics. Activists felt the effects of this pressure:

I mean I was terrified let me tell you, I was terrified of it. I was kind of ashamed of what I was doing in a way. Like, I thought, there were so many people campaigning against our little clinic in Galway, so many religious people and or semi-religious; the League of Decency and The Irish Family League and all of that sort of thing. But like, picketing the premises and you know, they would be outside the door every evening when I went there because I was working.^94^

Leaders in the movement also reported the application of personal religious pressure by families, employers, colleagues, and neighbours.

But while Catholicism generated normative pressures that shaped the precarity of the movement, it also provided unexpected opportunities for collaboration and mutual support. Former workers at the IFPA described the Association’s relationship with a Jesuit priest who would give absolution to Catholic women concerned that using contraception was a sin:^95^

[W]e’ve to send them round to this nice wee priest and he’d say, ‘No, God’s not like that. God’s not going to want you to leave your ten children and you’re not going to be excommunicated and I’ll give you forgiveness.’^96^

Another described the attitude of two nuns in St. Vincent’s Hospital to his talks on family planning: ‘Sr. Michael and I don’t approve of anything you stand for, but if you are having any more of those nights, you will invite us won’t you?’^97^ Sometimes, reinforcements from the Church were unexpected but quickly seized upon:

We had the opposition from the sources you’d expect. The church number one although as I say Bishop Lucey was our best advertisement. (…) Bishop Lucey very right wing, OK, that’s the generation he was and there was murder in Cork because suddenly this clinic selling these things has now opened and corrupting all the women you see. Anyway it opened and they were actually having, the business was very slow, the business was quite slow and they were quite concerned as to whether they could actually make a going concern of it until Bishop Lucey was so incensed by the opening of the clinic that he did a pastoral letter to be read out in every church in the dioceses on a Sunday morning condemning this disgusting and evil premises in Tuckey Street in Cork which was the vile contraceptive thing you see, well you couldn’t have got a better advertisement. The pastoral letter read out the address and Edgar Ritchie [founder of the Cork clinic] will tell you on the Monday he said after the Sunday he said there were nearly queues out the door.^98^

## Official Responses and the Effacement of Illegality

The necessary resulting plurality – perhaps hybridity – of the movement’s normative world means that its relationship with (and against) the law was sometimes clearly productive of its conditions, and at other times apparently insignificant. Rather than being primarily focused around the question of state enforcement, the movement’s persistence under conditions of fragile legality, was about complex conversations, or choreographies, between several orders that came to tolerate or discreetly undermine each other as time went by, as tactics were refined and contexts transformed.

A feature of our case study is the long-term maintenance of activities which broke or undermined the law in full view of the state. The Irish state’s prohibitions on condom distribution coexisted with an elaborate organized distribution network. The possibility of illegal distribution was an open secret. Nevertheless, it was only subject to limited, uneven, ambivalent interruptions by representatives of the state. For most of our interviewees, enforcement was a relatively insignificant background concern.

State responses to the movement were riddled with ambivalence. At one level, emanations of the state were openly critical of their activities. These activities needed monitoring by specialist police officers,^99^ were frequently condemned as immoral by officials, and the state was as reluctant to formally tolerate them as it was to legalize access to contraception. At other levels, state agents barely acted, although they did not lack opportunities to enforce the law.^100^ The failure to seize condoms is a case in point. Movements, places and actors were known. Condoms travelled through many spaces over which agents of the state had significant control before reaching customers. Yet these opportunities were not exploited. For example, postal services were rarely disrupted, and condoms almost always reached their purchasers. Asked if the post office ever seized condoms, one informant recalled:

I wouldn’t be surprised if it happened but we didn’t come across it and we didn’t have any nerves about it either. I mean they were fairly innocuous, brown envelopes with the person’s own writing. Now I suppose there would have been a rectangular shape in the packet. Yeah …^101^

Similarly, condoms passed by custom officers with rare disruptions. In part, this was due to the deployment of careful techniques of avoidance by those smuggling condoms over the border. However, even when briefly stopped, the condoms were always released and allowed, with their couriers, to continue their journey with no further state sanction than some wasted time and possibly awkward conversation. This story, told by one activist, was retold to us several times by others in the course of this research:

I had to import from London Rubber to import it down, from the North of Ireland, across the border and every second Saturday I would go to Portadown and it was my job to get through the border checkpoints, you see (…) and in those days you were stopped at the checkpoints, you are not now. And (…) I noticed they were stopping every third car. And I remember thinking ‘Oh fuck, I’m third’ and I had to change for another car, I would lift up the bonnet and then I would get through. And the only time I was ever stopped, I was way over the border, just outside Dublin in Balbriggan, you know where that is, it’s about 20 miles outside Dublin, and I was stopped at a Garda [police] checkpoint, they were looking for the IRA, they stopped me and I had 40,000 condoms. And the poor Guard didn’t know what to do. And he said ‘You are not supposed to have them on you.’ I said ‘Excuse me they are in my possession for my own personal use and I will challenge you if you interfere with me.’ And he just said ‘Have a nice weekend.’^102^

Other stories of interactions with custom officers illustrate further how skills and craft enabled condom smugglers to carefully avoid negative consequences. At the same time, they show the relative indifference of custom officers as state agents towards the circulation of contraceptives into Ireland:

This big Customs man comes down (…) and he looks at the car and he says ‘What have you got, what’s in the boxes?’ and I said ‘There is x thousand gross of Durex condoms’ and he looked at me and he looked and he said ‘Oh my God not on my shift, I don’t need this!’ (…) I said ‘I have all the documentation (…) There’s the forms, just stamp those, there is your cheque, let me off’ and he said ‘Do you know it’s half ten at night’ and I said ‘Just let me off’ and he said ‘I can’t, I have been reading things in the papers, I can’t.’ He said ‘Would you leave them in the office behind, would you leave them, just leave them.’ I said ‘Are you confiscating these?’ ‘Ah no, no’ he said, ‘Look (…), I’m on in the morning and I’ll have made a phone call.’ (…) I opened the boot and I had offloaded about a third of them and I thought ‘What am I doing, I’m like a sheep’ so I offloaded about half a dozen boxes, I had another 25 or something in the car, I slammed the boot, went into him and he said ‘OK’ and I said ‘That’s grand I’ll be in, in the morning’. ‘Good luck’ he said, I said ‘Bye’, gone. The following morning into FPS now down to their last condom you see. I felt like something out of the Wild West relieving the army or something. I rang the guy in the Customs and I said ‘How are you John?’ and he said ‘OK it’s OK Mr. [X] you can take them away’ he said ‘that’s fine.’ Then I went down you see and I said ‘Fine I’ll be down to pick them up’ and he said ‘I’m very worried.’ I said ‘What are you worried about?’, he said ‘I’m looking at the paperwork, you got 35 boxes and I can only count six.’ I said ‘John don’t you worry your little head about that. Don’t you just worry about it.’ He said ‘They’re not stolen?’ ‘No, no’ I said ‘just don’t worry about it’ and then he realised. ‘Oh’ he said ‘grand, that’s fine!’ I came down to pick up the six boxes, I gave him a couple of dozen for himself and that was it.^103^

Many interviewees reported encounters with individual agents that reached a degree of trust, intimacy, and civility. Official condemnation was commonly accompanied by more informal or friendly warnings, long-built relationships, limited enforcement:

We weren’t quite the enemy to be fair, there were a lot of people who would have been supporting us in the civil service, in the government but politically they couldn’t say so but you get a message back saying ‘Just be careful or you’ll fall.’^104^

These stories do not lend themselves to any clear overarching narrative of ‘the state’ as a unified entity. The state here is constituted through complex layers of micro-interactions that do not all go in a singular and uniform direction. Instead, relationships were made up of series of encounters and negotiations with individual agents, who can be disaggregated from the institutional state.^105^ A noticeable feature of these encounters is that (in)visibility mattered more than substance – a degree of discretion and of restraint was expected in return for state agents’ tolerance.

Other factors shaped individual agents’ behaviour. For several of our informants, the police were intimidated by or uncomfortable with having to engage with the family planning issue. As one former nurse put it, they were ‘kind of a bit afraid of us.’^106^ It is also important to bear in mind that state agents themselves used the family planning clinics’ services.^107^ For example, in 1984, when the police tried to bring an action against FPS’s Dún Laoghaire clinic, where staff were tricked into selling condoms to a plain-clothes officer, FPS’s lawyers wrote to the Commissioner of An Garda Siochána, openly acknowledging this ambivalent position:

Our client wishes to make it perfectly clear, through you, that no member of An Garda Siochána has permission to enter or remain on our client’s premises for any purpose with an actual or potential prosecution of our client, its Directors, its suppliers or employees, or the gathering, or obtaining by any means of evidence connected with such prosecution. This applies whether the members are in uniform or in plain clothes. Any apparent consent to the presence of members who attend for these purposes in plain clothes is obtained by deception and null and void. Our client’s attitude has always been set out above: it becomes necessary to state it in this formal way only because of a certain legal construction advanced by Counsel for the Prosecution in Dún Laoghaire Court. It is hardly necessary to add that members of an An Garda Siochána who attend in their private capacities and for their private and personal purposes are, as always, most welcome.^108^

Enforcement of the contraceptive laws was not inevitable, but negotiated at each level of interaction between individual, state agents, and formal expressions of state institutions.

In our case study, the ambivalence of these disaggregated state responses is complicated by the activists’ faith in the potential of state institutions.^109^ Indeed, they carefully used and engaged with the law throughout their activities. They hoped, after all, to carve a space for eventual legalized access to condoms, as a form of alterity to the dominant stance on family planning:

The ambition of the Family Planning movement as a movement … was to do ourselves out of business by ensuring that the state provided this service the way it does in the UK … There should be a state provided family planning service, there should be clinics like there were dispensaries years ago in every town and village in the country or at least in every major, so that people have access and free access to contraception.^110^

In time, it became clear that government was obliged to tolerate the movement, because it was effectively providing a well-supported highly visible public health service:

[The authorities] weren’t prepared to accept family planning clinics but they weren’t prepared to act against them because what was happening at that time was a huge ground-swell of change. It was those thirty-five year old women with three children, you know. If you took the clinic away from there, there was a serious constituency; do you know what I mean? And they knew the serious constituency and it wasn’t a radical left-wing group that you could close down. This was actually meeting a very real demand and these were regular people using the service and they weren’t prepared to touch it.^111^

Clearly, activists’ capacity for legal adaptation and improvisation was limited, and occasionally they would meet opposition. State practices of tolerance split open along lines that were only partly predictable. Enforcement was often targeted towards activists who were already otherwise vulnerable, such as women distributors in the radical feminist Contraception Action Programme,^112^ or otherwise subject to the criminal law, such as sex-workers.^113^ Instances of enforcement were also clustered around particular moments of heightened political tension about reproductive freedom. It is telling in this respect that family planning clinics were subject to intensified policing in the lead-up to the 1983 abortion referendum. In 1984 and 1985 attempts were made to prosecute both Well Woman and FPS^114^ for selling condoms. Conservative state agents may have felt newly empowered because of public antipathy towards family planning clinics suspected to be facilitating women’s access to abortion. This was something that some activists had anticipated – determining their clinics’ cautious approaches to abortion accordingly.^115^ Thus, activists’ relationships with agents of the state (when they encountered each other) fell into a careful choreography of the (un)acceptable, in which the relative power and legitimacy of their distribution projects shifted according to circumstance.

## Negotiating Non-Compliance, Anticipating Risk

In spite of its relatively rare materialization, fear of sanction was a common theme among interviewees. It varied from one person to the next:

I know I had to be really brave to do what I did at the times when I did, when I was younger, but I think I would have been quite intimidated by authority, like I say, the Gardaí coming, if they arrived at my door I probably would have dropped dead.^116^

For some, their confidence in negotiating legal restrictions actually increased as they became more experienced: ‘[A]s time went on I kind of found my feet a bit and I didn’t care any longer really, that was the truth.’^117^ Others were unafraid of the consequences from the start. One interviewee described the movement as ‘young people who really have no fear of the consequences, just do it because it’s right.’^118^ A number of our interviewees identified themselves as the type of people who took great pleasure in breaking the law for its own sake. Indeed, for many of this group, the motivation to remain involved in the movement waned once it became ‘legal’. They were quite keen, in particular, to draw the attention of state officials. But not everyone was willing to go so far. In particular, the IFPA was, at the time, considered more conservative in its approach to the law than FPS and its associated clinics:

[T]hey were trying to do it a little bit more by the book. We just thought, well we can just do it, we thought they were talking too much and, you know, we were not afraid to take the steps. And I suppose we didn’t know that much about the IFPA when we saw them as that kind of people, kind of a particular organisation … FPS were more sort of funky and just do it in the simplest way possible but just do it.^119^

Activists’ self-conception as providers of an important social service conditioned their responses to the fear of sanctions:

Now whether we really wanted to be carted off and brought to jail I don’t think it would have bothered me or others at the time if we had to do, it would have been part of the campaigning method but there would have been no risk involved, there would have been nobody’s health or risk involved.^120^

The Galway clinic members felt that the costs of that punishment to their clients might be greater than the costs to the FPS clinic in Dublin. This awareness constrained their law-breaking activities. They decided to ‘keep their heads down’, focus on service provision, and leave ‘politics’ to FPS:^121^

I mean, it was a real fear, it was a genuine fear and it was all very fine people saying … ‘Let them close you’ but what would happen to the people who were depending on your service? You had to be responsible at the same time whereas if they closed family planning services in Dublin, the IFPA was still there and the Well Woman was still there. If they closed the clinic in Galway look who you were affecting, Connemara, Mayo, all those people who had come to depend on that one little clinic.^122^

The project of anticipating the limits of state tolerance was always a ‘doing’; a makeshift composition,^123^ even as the infrastructure and networks become stronger and more stable. Activists moved strategically between carefully choreographed strategies of disobedience which would not expose the movement to backlash, and public radical challenge which might directly provoke enforcement of the law, either generating public support or exposing state powerlessness. As groups gained in confidence, and in experience of living with this situation, they could incrementally adjust the boundaries which illegality set to their projects by deciding to take new risks. They would decide how visible to make their illegality. Two examples illustrate the considerations in play.

The Health (Family Planning) Act 1979 came into force in November 1980. Its stated purpose was to ‘secure the orderly organisation of family planning services’, and this meant excluding and controlling established illegal distributors. Family planning services could be permitted, with the ‘consent’ of the Minister for Health, to provide family planning information and instruction, but not to sell or import contraceptives.^124^ Supply of contraceptives otherwise than by sale was banned.^125^ Most importantly, contraceptives could only be bought under the supervision of a pharmacist, with a doctor’s prescription. The doctor had to authorize the purchase having verified that the contraceptives would be used for ‘bona fide family planning purposes, or for adequate medical reasons’.^126^ Effectively, this legislation criminalized the clinic model. It also threatened the mail-order service – the funding engine of the clinics – since mail-order customers would not have obtained a prescription. More broadly, the legislation was a threat to the movement’s social mission. Condoms were much more expensive to purchase from a pharmacist than from the family planning clinic. By 1982, a packet of 12 condoms, available from FPS for £1.80,^127^ cost between £2.50 and £3.20 from a pharmacist.^128^ A doctor’s consultation usually cost £5 or £6 before any condoms were purchased.^129^ These charges placed contraceptive access well outside many working class clients’ reach. There was no health justification for requiring doctors to prescribe non-medical contraceptives.^130^ FPS determined that they would openly flout the law. They sold condoms without prescription, did not hire a pharmacist, and maintained the mail order service. Business ‘went on as normal’.^131^ Indeed, in 1984, FPS opened a new clinic which it publicly admitted would not be operating within the letter of the law.^132^ For the smaller rural Galway clinic, by contrast, compliance with the Act was next to impossible. They had no choice but to wait and see how the Act might be enforced.^133^ But FPS’s founders felt that they were in a strong enough position to live with imperfect compliance. As one founder explained: ‘[A]t that stage we were so well established it was a de facto achievement.’^134^

The decision not to comply was taken only after attempts at notional compliance with the law did not bear fruit. While the Act was still a Bill, FPS took legal advice on the possibilities for compliance with the law. Their barrister suggested that the Bill was possibly unconstitutional as ‘the state sets up the doctor as an arbiter of private morality for the family’.^135^ For a time, FPS considered finding three married couples to bring a constitutional challenge, but they were advised that they would have to wait to see how the Act operated in practice before they could assess the strength of the case. To challenge the Act, these couples would have to demonstrate that they were genuinely unable to access contraception.^136^ These constitutional arguments were of more use to FPS in critiquing the Act than in devising practical methods of resistance. FPS were also advised that vernacular tactics of legal interpretation would not work with this Act: ‘In so far as the regime being imposed is a strict one, there would seem to be little room for “liberal interpretation”.’^137^ Attempting to replicate their previous successful ‘literal’ approach to statutory interpretation, FPS suggested several ways in which the clinic could appear to comply with the Act without changing their basic model.^138^ They considered establishing a company, FPS Pharmacy Ltd., with a pharmacist who would occasionally visit shop space in some of the clinics, or contract with members of clinic staff as his ‘lay agents’. This would allow the clinic to operate as an extension of his business, selling on his behalf. They also proposed redesigning their mail-order form to include a declaration, stamped by a doctor, that the sale had been authorized under the 1979 Act ‘for the purposes of bona fide family planning’.^139^ But the legal advice received was that this tactic would fail: to comply with the Act all sales would have to be supervised directly by a pharmacist, doctors had no independent power of sale, and doctors would have to see clients in person.^140^ The redesign was abandoned.

FPS made one major change in response to the new law. In 1980, on solicitors’ advice, the wholesale distribution and importation part of FPS – Family Planning Distributors – was transferred to a new separate limited company, Dearsley Ltd.^141^ The same people sat on the boards of both companies. The intention, as their solicitors recognized, was to flout the law initially, and either seek an importation licence from the Minister in due course, or to query the constitutionality of aspects of the Act that would prevent them from importing and selling.^142^ FPS maintained that, while its distribution of condoms to consumers did not constitute ‘sale’, its wholesale activities probably did. In setting up the new entity which would operate outside the new law, they hoped to protect the supposedly ‘legal’ activities of the original company. They were also, however, taking risks: Dearsley might be tainted by association with FPS and might be refused a licence. However, an import licence was granted at the end of 1980, without any apparent query as to sale. In the years after the passage of the 1979 Act, therefore, FPS were once again in an ambivalent legal position. Writing to the Board in 1981, one FPS leader expressed some dissatisfaction with this situation: ‘We must clearly decide if we want to attempt to live within the law or alternatively come out clearly in defence of what we are doing.’^143^

By contrast, while the Act was still a Bill, FPS engaged in much less tentative complex disobedience along with members of Irishwomen United, in the Contraceptive Action Programme (CAP). Since 1976 CAP had engaged in sale as a form of direct action from stalls at locations like the Dandelion Market near Dublin’s Stephen’s Green, as well as in working-class areas of the city.^144^ In November 1978, CAP volunteers opened a temporary shop called Contraceptives Unlimited in a dilapidated building on Harcourt Road in Dublin to facilitate sales and to protest the imminent Health (Family Planning) Act 1979:

[I]t was a tiny little shop, it was almost like a kiosk, you know, with a table and a chair, I don’t think it had a phone or anything, and some boxes behind it, it was minimalist, and a variation of the price list that FPS was using was put in officially as a price list and put up on the wall basically, you know, these are the prices of these items, you know, a packet of condoms is so much. That was it, we just opened the door. I think we made up a sign and put it in the window, advertise it around.^145^

Around the same time, members of CAP in Cork were setting up stalls to sell or distribute condoms to working-class women and to raise awareness of the potential consequences of the 1979 Act. CAP was self-consciously more openly disobedient of the law than FPS: ‘[W]e were more radical, we were doing stuff that the nice middle-class people at family planning and the rest just didn’t do.’^146^ In part, this was possible because of the involvement of radical feminists, who had prior experience of audacious public disobedience, and were less interested – in this context at least – in strategic engagement with the status quo. But it was also possible because Contraceptives Unlimited completely decoupled the question of political protest both from service provision and commerce. There was no need for attention to profits. The shop kept a tiny stock and made few sales: ‘It was really a campaign, it wasn’t a shop.’^147^ Thus, FPS was protected from significant risk, which freed the shop for more audacious behaviour. ‘Sale’ here was sale and not donation: ‘[W]e just said, “Look, you know … we’re selling them, we’re not playing any games, we’re selling them”.’^148^ The main part of CAP’s ambition was to generate publicity,^149^ to draw attention to the particular provisions of law being broken, perhaps by provoking arrest:

And we went through scenarios and we said, ‘OK, anybody working in the shop must be agreeable and in a position to go to jail … [I]f it came to arrest, it was jail, because that was important as part of the agitation because the agitation was we are breaking the law, we are openly breaking your law and we want you to enforce it because we want the country and the world to see the stupidity of your law.’^150^

Arrest seemed a real possibility. Members of these branches saw their small stocks of condoms confiscated, and had uncomfortable encounters with the police.^151^ Desire to provoke imprisonment was rare among interviewees, and was a feature of specific planned actions such as Contraceptives Unlimited.^152^

## Condom Activism and ‘Illegality’

In this article, our interest is in the story of the Irish contraceptive movement as one about the social condition of ‘illegality’. The narratives and experiences of activists are rich in insights about what breaking the law – and breaking the law in order to change it – may mean. Activists told us stories of the *legal* agency and consciousness^153^ that shaped the survival and progression of the contraceptive movement. Illegality is more than a mere technical positioning ‘against’ the law. It is tempting to frame illegality as ‘a self-evident “fact”, generated by an act of violation’ of law.^154^ On that reading, the movement’s activities were illegal because they breached particular statutes, or interpreted them in ways which fell far outside official usage, and this was apparent in moments of public conflict with state authorities; seizures of goods, arrests, and prosecutions (even if, as we explained above, these were surprisingly rare). This static concept of ‘illegality’, however, may hide rather than recognize multiple practices that keep resistance activities in motion despite the text of state law. The stories in this article guide us away from this ‘fetishistic objectivity’,^155^ to see illegality as something inhabited in different ways from one interaction to the next.^156^ The experience of illegality in the Irish contraceptive movement was one of everyday disobedience, tactical adaptation, and forced improvisation.

Illegality is as much a matter of quotidian conditions and routine practices of perseverance as of ‘definitive events that occur in the world’.^157^ Stories of the Irish family planning movement show the significance of exploring illegality not only through exceptional or spectacular moments of conflict between law and disobedient ways of life but through more subtle processes of normalization and (un)settlement.^158^ Although we do not wish to ignore eventfulness, this study reminds us that much of what is important about illegality will often go on below that threshold.^159^ As Kristeva writes:

Modern revolt doesn’t necessarily take the form of a clash of prohibitions and transgressions that beckons the way to firm promises; modern revolt is in the form of trials, hesitations, learning as you go, making patient and lateral adjustments to an endlessly complex network.^160^

One interviewee aptly noted that ‘practical people’^161^ may also ‘test the law’:^162^

… we found a way around the law because if you go with the law head on I mean frankly the law has to win, it just has to win because otherwise society collapses. So there is no point trying to change something unless you are prepared to die with a gun in your hand which we weren’t. There is ways of changing the law and what you do is you find a way around it which actually suits all parties when you think about it.^163^

The history of Irish condoms activism also reminds us that what is displaced by law, unsaid, unacknowledged or prohibited in state law retains life elsewhere.^164^ Thus, against constructions of illegality as a state of total dispossession, the story of the Irish contraceptive movement shows that illegal practices can open up spaces for new exchanges and relationships, albeit uneven, fragmentary, or experimental as some are. Their long perseverance in illegality illustrates what it takes to strive in conditions of ‘stuckness’,^165^ of ‘making do’.^166^ Here we have found de Certeau’s conception of tactics useful to understand some of the movement’s activities. For him, a tactic:

operates in isolated actions, blow by blow. It takes advantage of ‘opportunities’ and depends upon them, being without any base where it could stockpile its winnings … What it wins, it cannot keep … It must vigilantly make use of the cracks … It poaches in them. It creates surprises in them … It is a guileful ruse.^167^

This is reflected in the contraceptive movement: they achieved a great deal in small-scale opportunistic engagement with law. To borrow from Elizabeth Povinelli, their history prompts us to think about what it means to ‘endure illegality’. Povinelli uses ‘endurance’^168^ to describe how alternative forms of social life ‘maintain the force of existing’.^169^ Attention must be paid to the ‘effort it takes to strive to persevere’^170^ in conditions of illegality.

Finally, the story of the contraceptive movement in Ireland reminds us of the significance of focusing on grass-roots activist practices of illegality and away from unified state-centric accounts of the legal/illegal divide. Law and society scholars are well aware of the blurriness of this divide, even though it often perseveres in broader public discourses. In turn, this case study enables us to reflect on the lived experience of negotiating this boundary: at one level, for the Irish contraceptive movement, it *mattered* that the law designated most of their activities as illegal. It meant that they needed to negotiate through asymmetrical power relations, where the dominant legal order maintained a certain (if challenged) hegemonic force. It generated a certain precarity which in turn shaped most activists’ day-to-day practices. It sustained a climate of fear and a sense that activities were always susceptible to external intervention or interruption. At the same time, activists’ stories reminded us that governmentality is dissipated and dispersed, so that the ways in which illegality is organized and experienced do not begin and end with state institutions (themselves complex entities constantly engaging with non-state orders). Thus, the experience of illegality is constituted by multiple sources of ordering, which rather than standing neatly alongside one another, are deeply entangled.^171^

In particular, the complexity of the relationship between state agents and activists in the context of ongoing illegality must be stressed. The maintenance of illegality was enabled by sets of careful negotiations, where state agents were to some extent willing to allow activists to act against the law. Those negotiations, however, were also inevitably tainted by the fact that state agents inhabited a more secure position as against the law: even when their projects were tolerated or facilitated by state agents, activists remained in a more fragile position than those that acted on behalf of the state. The frightening possibility that state agents would unilaterally interrupt an apparent fragile ‘settlement’ with the activists by suddenly enforcing the law in new ways was always present. As a result, illegality in the contraceptive access movement rarely took the form of direct and steady affront to powerful institutions. Instead, opportunities were seized as they came up, and previous strategies revisited when they ceased to be fruitful.

## Conclusion – From Endurance to Tranformation

The illegal activity of the family planning groups remained as a critical irritant to the legislation restricting contraceptive access, even after apparent law reform in 1979. Their illegal practices enacted critiques of the prevailing law; they made their own moral claims, and those of others, known through law breaking.^172^ They also established new Irish modes of engagement with contraception, not yet provided by the state, which were no longer saturated by religious morality or, necessarily, by conservative medical power, but instead were characterized by solidarity with clients, care and even humour. From a critical legal pluralist perspective, we could go further, and say that the illegal order established and sustained by the movement became, in itself, a ‘lived’ legal order more influential in many respects than the official law of the state.^173^ The story of the illegality of condoms in Ireland is one of a ‘plurality of resistances’^174^ that over time contributed to the construction of important durable social structures officially foreclosed by state law. The clinics’ activities became exercises in legal ‘world-making’.^175^ Though strictly illegal, sale of condoms was practically ‘licit’^176^ in the sense the movement’s activities were:

nonetheless perceived as a ‘normal order’, as licit activities insofar as they are admissible and have in some ways come to represent certain truths about the economic, or about ways of acting effectively in the economic realm.^177^

Repeated acts of disobedience by the movement and their clients, and refusal of full commitment to the state’s law weakened that law.^178^ Official determinations of the legal/illegal boundary was much less important than the public perception of where it lay, and than the activities surrounding it.

A necessary reciprocity of influence arose between the legal order and those causing trouble for it.^179^ Progressively, through tactical negotiation, the use of unexpected resources and creative resistance to multiple normative and practical pressures, the movement managed to establish itself as a more stable, more inevitable, socio-medical alternative space, that state institutions could not fully ignore or dislodge – and that they had little incentive to dislodge given its evident societal contributions. In doing so, the movement managed to rewrite legitimate possibilities for illegality. Eventually, the legal order had to adapt to the movement’s illegal practices, and the movement’s sale activities were fully legalized in 1992.^180^ However, the work of creating the new order must be distinguished from the work of legalizing it; the movement created a regime which was belatedly recognized in law. Indeed by the time the law fully recognized the movement’s activities, they had long moved on to condom-vending machines, which were not legalized until 1992. Understanding the process of ‘becoming legal’ requires (at least) an understanding of what it is to endure illegality in the first place. In this article, we have explained some of what was done by way of ‘maintaining the otherwise’,^181^ in the movement, accumulating and stabilizing ways of negotiating illegality which over time, became valid interpretations of the prevailing law, or independent legalities of their own.^182^

## Footnotes

1 In this article, ‘Irish’ and ‘Ireland’ refer to the Republic of Ireland.

2 Criminal Law Amendment Act 1935, s. 17. The advertising of contraceptives was also caught by censorship laws; see ss. 16(1) of the Censorship Act 1929 and s. 7 and s. 9(1) of the Censorship of Publications Act 1946. These sections were repealed by the Health (Family Planning) Act 1979.

3 See, further, U. Crowley and R. Kitchin, ‘Producing Decent Girls’ (2008) 15 *Gender, Place and Culture* 355; S. McAvoy, ‘Regulation of Sexuality in the Irish Free State’ in *Medicine, Disease and the State in Ireland, 1650-1940*, eds. G. Jones and E. Malcolm (1999) 254; S. McAvoy, ‘A Perpetual Nightmare: Women, Fertility Control, the Irish State and the 1935 Ban on Contraceptives’ in *Gender and Medicine in Ireland, 1700-1950*, ed. M.H. Preston (2012) 197.

4 Mary Robinson and colleagues made seven attempts to introduce a family planning bill: D. Ferriter, *Occasions of Sin: Sex and Society in Modern Ireland* (2009) 410.

5
*McGee* v. *AG* [1974] IR 284. For more detailed commentary on *McGee*, and subsequent legislative debates, see M. Enright and E. Cloatre, ‘Commentary on *McGee and AG*’ in *Northern/Irish Feminist Judgments*, eds. M. Enright et. al. (2017) 95.

6
*McGee* struck down s. 17(3) of the legislation, prohibiting importation. However, in the intervening six years, no law was passed to clarify the position on mass distribution of contraceptives.

7 See, for example, A. Beatty, ‘Irish Modernity and the Politics of Contraception, 1979-1993’ (2013) 17(3) *New Hibernia Rev.* 100.

8 Health (Family Planning) Act 1979, ss. 4(1) and (5).

9 id., s. 4(3).

10 Health (Family Planning) Amendment Act 1985.

11 The IFPA had been supplying contraceptives to clients for free, or advising them to import contraceptives in the post from the International Planned Parenthood Federation in England: M. Solomons, *Pro Life?: The Irish Question* (1992) 14.

12 See, for example, C. Hug, *The Politics of Sexual Morality in Ireland* (2016) 109-17; R.F. Foster, *Luck and the Irish: A Brief History of Change from 1970* (2008) 43; M.E. Daly, *Sixties Ireland: Reshaping the Economy, State and Society, 1957-1973* (2016) 148; Ferriter, op. cit., n. 4, p. 365.

13 For example, some founder members of FPS had also been members of the IFPA. They proposed mail-order distribution at the annual AGM but the motion was not successful, with some doctors who were active in the organization feeling strongly that distribution was going ‘too far’: RC, founder-member FPS; FC, founder-member FPS. One interviewee recalled that some doctors in the early Fertility Guidance Company were not keen on distributing contraceptives outside the clinic setting because ‘we wouldn’t like to get involved in trade’: RC, founder-member FPS.

14 On appearances of this binary in reproductive justice movements elsewhere, see R. Holz, ‘Nurse Gordon on Trial: those early days of the birth control clinic movement reconsidered’ (2005) 39 *J. of Social History* 112. It is important to note that very few informants articulated the kind of coherent radical anti-state legal consciousness associated with Fritzvold’s idea of ‘under the law’. In a different context, they might not have been perceived as radical at all: E.D. Fritzvold, ‘Under the Law: Legal Consciousness and Radical Environmental Activism’ (2009) 34 *Law & Social Inquiry* 799, at 810.

15 See, for example, L. Gordon, *The Moral Property of Women: a History of Birth Control Politics in America* (2007); A. Sarch, ‘Those Dirty Ads: Birth Control Advertising in the 1920s and ‘30s’ (1997) 14 *Critical Studies in Mass Communications* 31.

16 Both within literature on reproductive technologies (for example, L. Reagan *When Abortion was a Crime: Women, Medicine and Law in the United States 1867-1973* (1988)) and beyond (for example, N. De Genova, ‘Spectacles of Migrant “Illegality”: The Scene of Exclusion, the Obscene of Inclusion’ (2013) 36 *Ethnic and Racial Studies* 1180.

17 See, similarly, R.P. Fairbanks, *How It Works: Recovering Citizens in Post-Welfare Philadelphia* (2009) 190.

18 RC, founder-member FPS; FP, clinic volunteer IFPA; DM, clinic administrator, Galway; SMcA, client; CF, board member IFPA.

19 Y. Galligan, *Women and Politics in Contemporary Ireland: From the Margins to the Mainstream* (1998) 146. For a first-person account, see J. Levine, ‘From *Sisters: The Personal Story of an Irish Feminist* (1982)’ in *The Field Day Anthology of Irish Writing, Vol. 5*, ed. A. Bourke (2002) 200.

20 On the early days of the IFPA, see Solomons, op. cit., n. 11.

21 AMcC, founder-member FPS.

22 id.

23 DMcC, founder-member FPS.

24 RC, founder-member FPS.

25 DMcC, founder-member FPS; FC, founder-member FPS.

26 AMcC, founder-member FPS.

27 id.; DM, clinic administrator, Galway.

28 AMcC, id.

29 FC, founder-member FPS.

30 AMcC founder-member FPS; DMcC, founder member FPS.

31 RC, founder-member FPS.

32 DMcC, founder-member FPS.

33 RC, founder-member FPS.

34 DM, clinic administrator, Galway.

35 The Freedman archives contain extensive documentation of FPS’s commercial and governance activities post-1979. We could not find any similar resources for the period pre-1979.

36 SK, staff member IFPA.

37 DMcC, founder-member FPS.

38 AMcC, founder-member FPS.

39 PS, founder Galway clinic.

40 ES, founder Galway clinic.

41 id.

42 PS, founder Galway clinic.

43 ES, DM, BG, all involved in early Galway clinic.

44 D. Massey, *For Space* (2005) 140-2.

45 FC, founder-member FPS.

46 DMc, founder-member FPS; TO’B, staff member IFPA.

47 DM, clinic administrator, Galway.

48 FP, early volunteer IFPA; CD, staff member IFPA.

49 FP, id.

50 ES, founder Galway Clinic.

51 PS, founder Galway Clinic.

52 AC, director Well Woman; DM, clinic administrator, Galway.

53 DM id.

54 BG, early volunteer, Galway.

55 DM clinic administrator, Galway.

56 id.

57 N.L. Shier, ‘A History of the Limerick Family Planning Clinic 1975-1979’ (1999) MA dissertation, University of Limerick, 9.

58 AC, director, Well Woman. The Well Woman Clinic struggled to find doctors who would adhere to its feminist ethos, for example, by allowing women to read their own medical charts or by refusing to use stirrups.

59 AC, id.

60 R. Horning, ‘Precarity and “Affective Resistance”’ (2012) 1 *The New Inquiry*.

61 BA, nurse IFPA.

62 P. Ewick and S. Silbey, ‘Subversive Stories and Hegemonic Tales: Toward a Sociology of Narrative’ (1995) 29 *Law & Society Rev.* 197.

63 P. Ewick and S. Silbey, *The Common Place of Law: Stories from Everyday Life (1998) 136.*

64 For useful observations on using law to secure illegal activity, see L. Finchett-Maddock, *Protest, Property and the Commons: Performances of Law and Resistance* (2016) 66, 101.

65 RC, founder-member FPS.

66 id.

67 DMcC, founder-member FPS.

68 RC, founder-member FPS.

69 UCD Stydents’ Union Archive, ‘Provision of Contraception’ (2015), at <http://su40.ucd.ie/provision-of-contraception/>.

70 PS, founder Galway clinic.

71 ES, founder Galway clinic.

72
*McGee*, op. cit., n. 5.

73 RC, founder-member FPS.

74 See, for example, M. Strathern, ‘Gifts Money Cannot Buy’ (2012) 20 *Social Anthropology* 397.

75 P. Ewick and S. Silbey, ‘Narrating Social Structure: Stories of Resistance to Legal Authority’ (2003) 108 *Am. J. of Sociology* 1328.

76 NS, nurse IFPA.

77 AC, director Well Woman.

78 AMcC, founder-member FPS.

79 DMcC, founder-member FPS.

80 RC, founder-member FPS.

81 Barrister’s Opinion, 9 September 1980: Freedman archive, UCD.

82 Fairbanks, op. cit., n. 17, p. 191.

83 DM, clinic administrator, Galway.

84 AC, director Well Woman.

85 RC, founder-member FPS.

86 ES, founder Galway clinic; CD, staff member IFPA.

87 For further reflections on activist deployments of a sense of formal law’s illegitimacy, see Finchett-Maddock, op. cit., n. 64; S. Halliday and B. Morgan, ‘I Fought the Law and the Law Won? Legal Consciousness and the Critical Imagination’ (2013) 66 *Current Legal Problems* 1.

88 DMcC, founder-member FPS.

89 id.

90 ES, BG, LS, all female interviewees.

91 FP, early volunteer IFPA.

92 GS, member CAP.

93 MF, member CAP.

94 DM, clinic administrator, Galway.

95 NS; CD; SK, staff members IFPA.

96 CD, id.

97 FC, founder-member FPS.

98 AMc, founder-member FPS.

99 FC, founder-member FPS.

100 Our focus here is on everyday enforcement rather than litigation. However, we should note that one criminal prosecution was brought against FPS and the IFPA in 1974 on the evidence of the Catholic activist John O’Reilly, who had ordered contraceptives and a family planning booklet from FPS and the IFPA. His young daughters had signed the order letters. He argued that FPS and the IFPA had breached both the criminal prohibition on sale of contraceptives, and the provisions of the censorship legislation which prohibited the sale or distribution of obscene literature: a family planning booklet. Kearney J. in the District Court found against him on both counts. In particular, he found that there was no evidence that FPS had illegally sold contraceptives: ‘Family plan case’ *Irish Independent*, 20 February 1974. The prospect of prosecution did not have a chilling effect on FPS’s activities. While the court did not confirm the legality of the sale/donation tactic, FPS welcomed the publicity generated by the case. They were emboldened by this success and felt reassured that they would not be subjected to a similar challenge again: DMcC, founder-member FPS. One interviewee expressed regret that they had won the O’Reilly case, since imprisonment of activists would have generated significant publicity for the movement: RC, founder-member FPS. When the family-planning booklet was subsequently censored, the IFPA successfully challenged the ban in the High Court and were again successful when the Attorney General appealed to the Supreme Court: *Irish Family Planning* v. *Ryan* [1979] IR 295.

101 ES, founder Galway clinic.

102 FC, founder-member FPS.

103 AMcC, founder-member FPS.

104 id.

105 Ewick and Silbey, op. cit, n. 75.

106 NS and BA, IFPA nurses.

107 CD, IFPA staff; AC, director Well Woman.

108
*Director of Public Prosecution* v. *Oonagh McCutcheon* – Letter from Hussey & Co., solicitors to FPS, 12 July 1985: Freedman Archive UCD.

109 On complexity and contradiction in activist legal consciousness, see Fritzvold, op. cit., n. 14.

110 DM, administrator Galway clinic. For discussion of similar tensions in a British context, see A. Leathard, *The Fight for Family Planning* (1980).

111 PS, founder Galway clinic.

112 LS, GS, members CAP

113 MK, former sex-worker and activist, Dublin.

114 Hussey & Co., op. cit., n. 108.

115 ES, founder Galway clinic; DMcC, founder-member FPS; RC, founder-member FPS.

116 SK, staff member IFPA.

117 DM, clinic administrator, Galway.

118 AMcC, founder-member FPS.

119 ES, founder Galway clinic.

120 AC, director Well Woman.

121 JW, clinic doctor, Galway.

122 DM, clinic administrator, Galway.

123 A. Vasudevan, ‘The Makeshift City: Towards a Global Geography of Squatting’ (2014) 39 *Progress in Human Geography* 338.

124 Health (Family Planning) Act 1979, s. 3.

125 id., s. 4(4).

126 id. ‘For adequate medical reasons’ regularized the established practice of prescribing the pill for non-contraceptive purposes.

127 FPS mail order form 1979: Freedman Archive UCD.

128 Letter, 9 February 1982; Association of Irish Family Planning Clinics (AIFPC), ‘Critique of the 1978 Bill’, 12 February 1979: Freedman Archive UCD.

129 Letter, 28 April 1981: Freedman Archive UCD.

130 AIFPC, op. cit., n. 128.

131 RC, founder-member FPS.

132 ‘Minister opens “law-breaking” family planning clinic’ *Evening Herald*, 16 April 1984: Freedman Archive UCD.

133 DM, clinic administrator, Galway.

134 DMcC, founder-member FPS.

135 Barrister’s Opinion, 7 January 1979: Freedman Archive UCD.

136 Meeting, August 1980: Freedman Archive UCD.

137 Opinion, op. cit., n. 81.

138 Case for counsel to advise – from Goodbody’s solicitors on behalf of FPS Ltd.; Meeting, 15 July 1980; Questions to Counsel, 21 August 1980: Freedman Archive UCD.

139 FPS, op. cit., n. 127. Doctors prescribing contraception under the Act had to endorse the prescription with a similar statement; Health (Family Planning) Act 1979, s. 4(1)(b)(ii).

140 Opinion, op. cit., n. 81.

141 Letter from Goodbody’s solicitors, 12 June 1980: Freedman Archive UCD.

142 id.

143 Letter to the board 12 December 1981: Freedman Archive UCD.

144 GS, LS, members CAP.

145 RC, founder-member FPS.

146 TO’K, member CAP.

147 SK, staff member IFPA.

148 TO’K, member CAP.

149 GS, member CAP.

150 TO’K, member CAP.

151 LS, GS, members CAP.

152 ES, founder Galway clinic; PS, founded Galway clinic; SK, staff member IFPA; CD, staff member IFPA.

153 D. Cowan, ‘Legal Consciousness: Some Observations’ (2004) 67 *Modern Law Rev.* 928; S.S. Silbey, ‘After Legal Consciousness’ (2005) 1 *Ann. Rev. of Law and Social Science* 323; P. Ewick and S.S. Silbey, ‘Conformity, Contestation, and Resistance: An Account of Legal Consciousness’ (1991) 26 *New England Law Rev.* 731.

154 De Genova, op. cit., n. 16.

155 id.

156 J.S. Anjaria, ‘Ordinary states: Everyday corruption and the politics of space in Mumbai’ (2011) 38 *Am. Ethnologist* 58.

157 E.A Povinelli, *Economies of Abandonment: Social Belonging and Endurance in Late Liberalism* (2011) 10.

158 B. Latour, *Reassembling the Social: An Introduction to Actor-Network-Theory* (2005); B. Latour, *We Have Never Been Modern* (1993).

159 Povinelli, op. cit., n. 157, p. 32; L. McNay, *The Misguided Search for the Political* (2014); L. Berlant, ‘Slow Death (Sovereignty, Obesity, Lateral Agency)’ (2007) 33 *Critical Inquiry* 754.

160 J. Kristeva, *Revolt, She Said* (2002) 54.

161 DM, clinic administrator, Galway.

162 DMcC, founder-member FPS.

163 AMcC, founder-member FPS.

164 D. Delaney, ‘Tracing Displacements: Or Evictions in the Nomosphere’ (2004) 22 *Environment and Planning D: Society and Space* 847, at 852.

165 S.B. Coutin, ‘Being En Route’ (2005) 107 *Am. Anthropologist* 195, at 196.

166 Ewick and Silbey, op. cit., n. 62.

167 M. de Certeau, *The Practice of Everyday Life*, tr. S. Rendall (2011) 37.

168 Povinelli, op. cit., n. 157. See, also, the idea of survival in K. Peterson, *Speculative Markets: Drug Circuits and Derivative Life in Nigeria* (2014).

169 Povinelli, id., p. 9.

170 id.

171 N. Blomley, ‘Un-Real Estate: Proprietary Space and Public Gardening’ (2004) 36 *Antipode* 614, at 621; M. Nuijten, ‘Illegal Practices and the Re-Enchantment of Governmental Techniques’ (2003) 35 *J. of Legal Pluralism and Unofficial Law* 163.

172 See, for example, Fritzvold, op. cit., n. 14.

173 M. Davies, ‘Feminism and the Flat Law Theory’ (2008) 16 *Feminist Legal Studies* 281, at 301.

174 ‘[T]here is no single locus of great Refusal, no soul of revolt, source of all rebellions, or pure law of the revolutionary. Instead there is a plurality of resistances, each of them a special case …’ M. Foucault, *The History of Sexuality: Vol. 1, An Introduction* (1990) 95–6.

175 Ewick and Silbey, op. cit., n. 75.

176 For further insights into the interface between ‘illegal’ and ‘licit’ activities, see J. Roitman, ‘The Ethics of Illegality in the Chad Basin’ in *Law and Disorder in the Postcolony*, eds. J. Comaroff and J. Comaroff (2006) 247.

177 id., p. 264.

178 If performance of obedience is what gives law purchase in the material world, then the Irish contraceptive laws were arguably stripped of obligatory force by repeated disobedience: E. Melissaris, *Ubiquitous Law: Legal Theory and the Space for Legal Pluralism* (2016) 41; H. Lindahl, *Fault Lines of Globalization: Legal Order and the Politics of A-Legality* (2013) 118.

179 Ewick and Silbey, op. cit., n. 75.

180 Health Family Planning (Amendment) Act 1985.

181 Povinelli, op. cit., n. 157.

182 J. Etxabe, ‘The Legal Universe After Robert Cover’ (2010) 4 *Law and Humanities* 115, at 116. We can draw a useful analogy with Ewick and Silbey’s ‘legal consciousness’ which is: part of a reciprocal process in which the meanings given by individuals to their world become patterned, stabilised and objectified. These meanings, once institutionalised, become part of the material and discursive systems that limit and constrain future meaning making. (Ewick and Silbey, op. cit., n. 63, pp. 29-30.) See, also, Melissaris, op. cit., n. 178, pp. 82, 151.

